# Getting quantitative on the effects of somatic mutation on cancer

**DOI:** 10.18632/oncoscience.521

**Published:** 2020-10-27

**Authors:** Jeffrey P. Townsend

**Affiliations:** ^1^Department of Biostatistics, Yale School of Public Health, New Haven, CT, USA

**Keywords:** cancer genetics, prevalence, frequency, somatic variant, natural selection, effect size, therapeutic development

Numerous powerful bioinformatic analyses of cancer tumor sequencing have applied sophisticated mutation calling, determining the key cancer-causing variants and quantifying their prevalence. The calculations of prevalence of a mutation across tumors and the determination of the statistical significance of whether it is a driver are the “shoulders” that have enabled the build-up to the most useful metrics about cancer variants—metrics which quantify the effect of the variant on replication and survival of the cancer lineage. Ostrow et al [1] effectively and comprehensively applied ratios of non-synonymous change to synonymous change to quantify natural selection in the somatic evolution of cancer, an approach that has been followed by others in different ways and contexts since then [2–4]. Martincorena et al 2 performed a cogent gene-wide analysis using mutation signatures c.f. [5] on the larger data sets available three years later.

More recently, it was revealed that previous studies have reported variant prevalence and P value, but have not reported cancer effect sizes, which quantify the effect of natural selection on somatic mutations within cancer cell lineages. Deconvolving the baseline variant mutation rates enables estimation of the selection intensity of individual mutations [6, 7]—a quantification that should be directly interpreted as a cancer lineage effect size that should be used in decision-making. This measure of the effect of specific somatic mutations on cancer cell proliferation and survival should be widely appreciated as a primary consideration of precision-medicine tumor boards, which are in operation at hospitals around the world. Effect sizes of somatic mutations should also be a key consideration in the initiation and design of precision medicine clinical trials: the number of trials has been increasing so rapidly that some have argued that demand is vastly outpacing the supply of enrollable patients [8]. Effect sizes should guide target selection in pharmacological development, an approximately three billion dollar industry [9]. And gene-specific site-specific effect sizes should guide basic research prioritization toward those important components of molecular and cell biology that have long-term potential to lead to therapies and cures for cancer.

There appear to be increasing numbers of drivers in each cancer as we examine larger and larger datasets [10], and each driver has its own quantitative effect on cancer [11]. Decoupling the contributions of mutation and cancer lineage selection to the frequency of somatic variants among tumors is critical to understanding—and predicting—the therapeutic potential of different interventions [12]. Importantly, antagonistic and synergistic epistasis among mutations also impacts the potential therapeutic benefit of targeted drug development [13]. Active use of these quantitative approaches are essential to furthering basic research on cancer, informing clinical practice, and providing rigorous guidance regarding investment in targeted drug development. By integrating evolutionary principles and detailed mechanistic knowledge into those approaches, we can maximize our ability to apply and develop targeted therapies that are likely to yield substantial clinical benefit
